# Estimating the Prevalence of and Clarifying Factors Associated With Multiple Tobacco Product Use in Japan: A Cross-sectional Study in 2022

**DOI:** 10.2188/jea.JE20240153

**Published:** 2025-05-05

**Authors:** Takafumi Yamamoto, Hazem Abbas, Upul Cooray, Tetsuji Yokoyama, Takahiro Tabuchi

**Affiliations:** 1 Preventive Dentistry, Hokkaido University Hospital, Hokkaido, Japan; 2 Department of International and Community Oral Health, Tohoku University, Graduate School of Dentistry, Miyagi, Japan; 3 Promotion Office for Inter-University Exchange Project, Division for Globalization Initiative, Tohoku University, Graduate School of Dentistry, Miyagi, Japan; 4 National Dental Research Institute Singapore, National Dental Centre Singapore, Singapore; 5 Department of Health Promotion, National Institute of Public Health, Saitama, Japan; 6 Department of Cancer Control Center, Osaka International Cancer Institute, Osaka, Japan; 7 Tohoku University School of Medicine, Division of Epidemiology, Department of Health Informatics and Public Health, School of Public Health, Miyagi, Japan

**Keywords:** multiple tobacco product use, cigarettes, heated tobacco products, prevalence, Japan

## Abstract

**Background:**

Multiple tobacco product (MTP) use is a public health concern due to their combined adverse health effects. MTP use may have increased since heated tobacco products (HTPs) became more prevalent in Japan. This study aimed to (1) estimate the recent prevalence of MTP use and clarify the associated factors compared to (2) non-smokers and (3) single-product users.

**Methods:**

We used data from an internet survey conducted in February 2022. The prevalence of MTP use in Japan was estimated using inverse probability-weighted scores from this survey and a nationwide survey by the Japanese government. Tobacco products include six types: cigarettes, HTPs, e-cigarettes, cigars, pipe/water pipes, and smokeless tobacco products. MTP use was defined using the outcome variable (no use, single-product use, and MTP use) based on these six types of use. Using multivariate logistic regression, we calculated the adjusted odds ratios and 95% confidence intervals (CI) to clarify factors associated with MTP use compared to non-smokers, adjusting demographic variables, psychological distress, self-rated health, and alcohol use. Using multivariate Poisson regression, we calculated the adjusted prevalence ratios (aPRs) and 95% CIs to clarify factors associated with MTP use among smokers, adjusting for these covariates and smoking-related factors like workplace and home smoking rules.

**Results:**

We analyzed 30,141 participants whose mean age was 47.8 years (standard deviation, 17.9), and 14,722 participants were male (48.8%). The estimated prevalence of MTP use was 6.8%. The most common combination of MTP use was cigarettes and HTPs. Compared to non-smokers, being younger, male, alcohol drinkers, and having low education, poor psychological distress, and poor self-rated health were factors associated with MTP use. Among smokers, workplace smoking rules, such as a partial smoking ban and no smoking ban, were not associated with MTP use compared to the indoor smoking ban. However, having no home smoking ban was positively associated with MTP use compared with a ban on both cigarettes and HTPs at home (both cigarettes and HTPs allowed aPR 1.36; 95% CI, 1.15–1.61, HTPs only allowed aPR 1.73; 95% CI, 1.43–2.10).

**Conclusion:**

MTP users may account for a high percentage of Japanese smokers.

## INTRODUCTION

Cigarette smoking continues to be a major cause of death throughout the world.^1^ While the use of cigarettes is decreasing,^2^^,^^3^ the use of novel tobacco products, such as heated tobacco products (HTPs) and electronic cigarettes (e-cigarettes), has been rapidly increasing, mainly due to tobacco industry marketing.^4^^,^^5^ Many new tobacco products have continued to enter the market annually, exposing smokers to numerous opportunities to use multiple tobacco products (MTPs). The use of MTPs has become a major concern in several countries due to the negative health impacts of tobacco use.^6^^–^^8^ MTP users have a greater risk of nicotine dependence and are more likely to continue tobacco use over time than single-product users.^9^

The prevalence of Japanese MTP use in 2017 was 3.2%^10^; however, this occurred during the early years of HTP availability in the market. The number of HTP users in Japan has continued to increase since 2015, when HTPs were first introduced to the market. The total sales volume of HTPs rose by 12.0% from 2020 to 2021.^11^ Several studies have indicated that most HTP users are dual users (ie, MTP users).^12^^,^^13^ Therefore, the number of MTP users may have increased in Japan since 2017.

Few studies have focused on the prevalence of MTP use and associated factors of MTP use since HTPs became widely available in Japan. This study aimed to (1) estimate the prevalence of MTP use in 2022; (2) clarify the factors associated with MTP users and single-product users compared to non-smokers; and (3) clarify the associated factors of MTP use among smokers.

## METHODS

### Study design, population, and data collection

We used data from a cross-sectional survey conducted between February 1 and February 28, 2022 for the Japanese ‘Society and New Tobacco’ Internet Survey (JASTIS 2022 study).

The JASTIS 2022 survey recruited participants from a pool of over 2 million panelists at Rakuten Insight, a major Internet research company, to represent all social categories. Participants were randomly selected to eliminate bias based on region, gender, or age. All panelists registered with Rakuten agreed to participate in various research surveys and provided written informed consent via the web. For minors, we obtained consent and digital approval from a parent or guardian before conducting the survey.

To avoid reporting bias, we excluded respondents who provided incorrect or inconsistent answers^14^: those who responded incorrectly when asked to choose the second option from the bottom (*n* = 2,676) in a dummy question, those who answered ‘yes’ to every option in a set of questions about the drugs they took (*n* = 155), and those who answered ‘yes’ to every option in a set of questions about the chronic diseases they had (*n* = 28). Although the legal smoking age in Japan is 20 years, we did not restrict the age of study participants to 20 years and older based on a previous study that estimated the prevalence of the use of various tobacco products across Japan.^10^ Participation in the survey was voluntary, but participants were required to answer all questions to complete the survey. As a result, there was no missing information in our survey data.

To achieve the three aims of this study, we identified two groups of respondents for analysis. First, to estimate the prevalence of MTP use in Japan, all respondents (non-smokers, single-product users, MTP users) were included. Second, to identify the factors associated with single-product users and MTP users compared to non-smokers, all respondents (non-smokers, single-product users, MTP users) were also included. Finally, to identify the factors associated with MTP users compared to single-product users, we narrowed down the respondents to only those who were classified as smokers (single-product users, MTP users).

### Measurement: current tobacco and tobacco-like product use

Current tobacco and tobacco-like product use was determined by asking: “Within the last month have you used the following products?” The options were “factory-made cigarettes”, “roll-your-own cigarettes”, “Ploom TECH”, “Ploom S”, “Ploom X”, “IQOS”, “glo”, “lil HYBRID”, “nicotine e-cigarette”, “non-nicotine e-cigarettes”, “e-cigarettes with unknown nicotine content”, “cigars”, “pipe”, “kiseru (Japanese pipe)”, “chewing tobacco”, “snu”, and “water pipe (hookah)”.

Type of tobacco use was defined as six kinds of tobacco products (cigarettes, HTPs, e-cigarettes, cigars, pipe/water pipes, and smokeless tobacco products) based on previous studies.^15^^,^^16^ For each type of tobacco product, participants were asked to select one of four options: never use, quit, use occasionally but not every day, and use almost every day. These four options were then grouped into two categories: those who do not use (never use, quit) and those who do use (use occasionally but not every day, use almost every day). Current HTPs users were defined as those who reported using the HTPs identified in the survey, including those available in Japan at the time of the study (Ploom Tech, Ploom Tech+, Ploom S, IQOS, glo, glo sens, Pulze). Current e-cigarette users were defined as those who reported using any type of e-cigarette (with or without nicotine and unknown nicotine content). Current pipe/water pipe tobacco users were defined as those who reported using a pipe, kiseru, or water pipe. Current smokeless tobacco users were defined as those who reported using chewing tobacco or snu.

Among current users of each product, those who reported using only one product were categorized as single-product users. Those who reported using multiple products were categorized as MTP users (using two to six products). Finally, MTP use was categorized into three groups: no current product use, single-product use, and MTP use.

### Measurement: covariates

Based on a review article focusing on the conceptual framework for MTP use,^9^ the following variables were used as covariates: factors indirectly related to smoking (age, sex, educational attainment, psychological distress, self-rated health, and alcohol use) and factors directly related to smoking (living with a smoker, exposure to tobacco company advertising in the last 6 months, current smoking rules in the workplace, and current smoking rules at home). However, factors directly related to smoking were not included in the model for the analysis aimed at identifying the characteristics of MTP users and single-product users compared to non-smokers.

The categories of each variable were as follows: age (17–29, 30–39, 40–49, 50–59, 60–69, 70–81 years), sex (female, male), educational attainment (2 years of college or more, high school or less), psychological distress (none, moderate, severe), self-rated health (good [excellent/very good/good] or poor [fair/poor]), alcohol use (no/quit, less than twice a week, more than twice a week), living with a smoker (no, yes), exposure to tobacco company advertising in the last 6 months (no, yes), current smoking rules in the workplace (indoor smoking ban, partial smoking ban, no smoking ban, do not know), and current smoking rules at home (both cigarettes and HTPs prohibited, both cigarettes and HTPs allowed, HTPs only allowed, do not know).

### Statistical analyses

First, we calculated descriptive statistics on the use of each smoking product among participants. We also attempted to estimate the use of smoking products by smokers all over Japan using inverse probability-weighted scores^17^ calculated from a nationally representative cross-sectional survey (ie, the Comprehensive Survey of Living Conditions of People on Health and Welfare [CSLCPHW] conducted by the Japanese Ministry of Health, Labour and Welfare in 2016) because the results of the Internet survey have limited generalizability due to the presence of selection bias.^18^ The inverse probability-weighted score was calculated based on the probability of being an ‘internet survey respondent’ (ie, propensity score estimated using a logistic regression model). This model included demographic and socioeconomic factors, such as education and housing tenure, using data pooled from two surveys (JASTIS and CSLCPHW). Additionally, to understand the prevalence of the major tobacco products in Japan (ie, cigarettes and HTPs), we also calculated descriptive statistics for smokers who exclusively use either cigarettes or HTPs.

Next, using the weighted scores, multivariate logistic regression was conducted to identify factors associated with single-product users and MTP users compared to non-smokers. We calculated the adjusted odds ratios (aORs) and 95% confidence intervals (CIs) with adjustment for covariates included in the model. Third, using the weighted scores, Poisson regression analysis was conducted to identify factors associated with MTP users compared to single-product users. We calculated the adjusted prevalence ratios (aPRs) and 95% CIs with adjustment for covariates included in the analysis model. The Poisson regression model was selected, as the prevalence of MTP users was high (>30%) in the smokers group. Non-smokers were excluded from the analysis to determine whether MTP users have different characteristics from single-product users.

All analyses were performed using Stata software (version 16.1; StataCorp LP, College Station, TX, USA). The threshold for significance was set at *P* < 0.05, two-tailed.

## RESULTS

**Figure 1.**
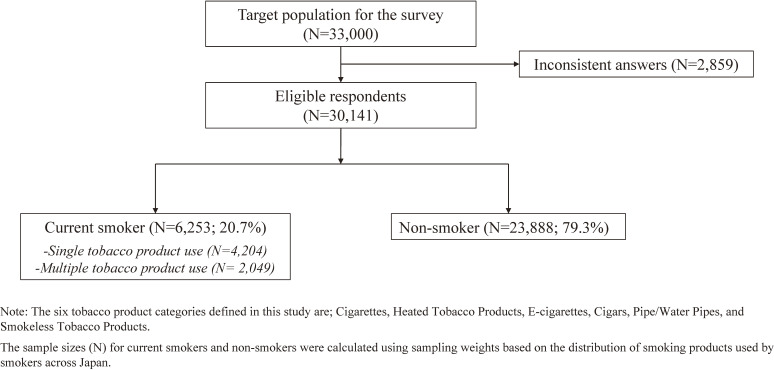
Flow-chart of this study

Figure 1 shows a flowchart of the participants in this survey. Of the 33,000 respondents approached, those who answered inconsistently were excluded from the analysis (*n* = 2,859), leaving 30,141 participants. We show the characteristics of study participants in Table 1. After adjusting for sampling weights based on the distribution of smoking products used by smokers across Japan, the mean age was 47.8 (standard deviation, 17.9) years, and 48.8% were male. Of these, 23,888 (79.3%) were non-smokers, while 6,253 (20.7%) were smokers, comprising 4,204 (13.9%) single-product users, and 2,049 (6.8%) MTP users (dual or more).

**Table 1.** Characteristics of study participants (*N* = 30,141)

		No Current Product Use (Non-smokers)	Total Product Use (Tobacco and Tobacco-like Products Use) (Current smokers)		*P* ^a^
		(Weighted *N* = 23,888; 79.3%)	Single-product use (Weighted *N* = 4,204; 13.9%)	MTP use (Weighted *N* = 2,049; 6.8%)	
		*N* (%)	*N* (%)	*N* (%)	
Age, years	17–29	5,592 (82.8)	575 (8.5)	584 (8.7)	**<0.001**
	30–39	2,866 (70.4)	804 (19.8)	399 (9.8)	
	40–49	3,824 (71.8)	1,039 (19.5)	463 (8.7)	
	50–59	3,479 (75.3)	847 (18.3)	294 (6.4)	
	60–69	3,944 (83.0)	572 (12.0)	237 (5.00)	
	70–81	4,182 (90.5)	367 (7.9)	73 (1.6)	
Sex	Female	13,808 (89.6)	1,219 (7.9)	392 (2.5)	**<0.001**
	Male	10,079 (68.5)	2,986 (20.3)	1,657 (11.2)	
Educational attainment	2 years of college or more	12,448 (77.7)	2,404 (15.0)	1,176 (7.3)	**<0.001**
	High school or less	11,440 (81.0)	1,800 (12.8)	873 (6.2)	
Psychological distress	None	13,061 (80.4)	2,324 (14.3)	857 (5.3)	
	Moderate	5,171 (81.2)	848 (13.3)	347 (5.5)	**<0.001**
	Severe	5,656 (75.1)	1,032 (13.7)	845 (11.2)	
Self-rated health	Good	21,005 (79.5)	3,667 (13.9)	1,760 (6.6)	
	Poor	2,882 (77.7)	538 (14.5)	289 (7.8)	**<0.001**
Alcohol use	No/quit	9,343 (86.1)	1,122 (10.3)	393 (3.6)	
	<2 times/week	7,874 (80.3)	1,261 (12.9)	665 (6.8)	**<0.001**
	≥2 times/week	6,671 (70.4)	1,821 (19.2)	990 (10.4)	

HTPs, heated tobacco products; MTP, multiple tobacco product; *P*, *P*-value.

All *N* and % were calculated using sampling weight from smoking products by smokers all over Japan. All weighted values were rounded.

A single-product user refers to a smoker who uses only one of the following tobacco products: cigarettes, heated tobacco products, e-cigarettes, cigars, pipe/water pipes, and smokeless tobacco products.

Please refer to Table 4 for the descriptive statistics of factors directly related to smoking (living with a smoker, exposure to tobacco company advertising in the last 6 months, current smoking rules in the workplace, and current smoking rules at home).

All weighted values were rounded.

^a^χ2 test.

We show the estimated prevalence of tobacco product use in 2022 using weighted scores in Table 2. Among dual users, the most prevalent types of tobacco product were cigarettes and HTPs, with an estimated prevalence of 3.2% in the Japanese population. Additionally, 14.0% of the population were estimated to use at least cigarettes. Of these, 5.3% were MTP users. Concurrently, 10.3% of the population in Japan were estimated to use at least HTPs. Of these, 5.6% were MTP users.

**Table 2.** Prevalence of total, single, and multiple current product use among study participants in Japan, 2022 (*N* = 30,141)

	*N*	Weighted *N*	Weighted % (95% CI)^b^
Total Product Use^a^ (Tobacco and Tobacco-like Products Use) (Current smokers)	5,519	6,253.0	20.7 (20.0–21.5)
Single Product Use	3,741	4,204	13.9 (13.4–14.5)
Multiple Product Use (two products or more)	1,778	2,049	6.8 (6.3–7.3)
Dual Product Use	1,210	1,439	4.8 (4.3–5.2)
Poly Product Use (three products or more)	568	610.4	2.0 (1.7–2.3)
No Current Product Use (non-smokers)	24,622	23,888.0	79.3 (77.8–80.6)

Single Products Use			

Cigarettes^c^	2,344	2,619.0	8.7 (8.1–9.3)
HTPs^d^	1,196	1,403.0	4.7 (4.3–5.1)
E-cigarettes^e^	87	88.1	0.3 (0.2–0.4)
Cigars	56	48.4	0.2 (0.1–0.2)
Pipe/Water Pipes	40	30.7	0.1 (0.04–0.2)
Smokeless Tobacco Products^f^	18	14.9	0.05 (0.01–0.08)

Dual Products Use			

Cigarettes and HTPs	798	952.3	3.2 (2.8–3.5)
Cigarettes and E-cigarettes	69	69.4	0.2 (0.1–0.3)
Cigarettes and Cigars	114	153.3	0.5 (0.4–0.7)
Cigarettes and Pipe/Water Pipes	18	13.7	0.04 (0.01–0.07)
Cigarettes and Smokeless Tobacco Products	6	5.4	0.01 (0.00–0.07)
HTPs and E-cigarettes	126	130.6	0.4 (0.3–0.6)
HTPs and Cigars	43	27.1	0.08 (0.04–0.13)
HTPs and Pipe/Water Pipes	42	24.2	0.08 (0.04–0.11)
HTPs and Smokeless Tobacco Products	37	31.3	0.10 (0.01–0.19)
E-cigarettes and Cigars	29	10.8	0.03 (0.01–0.05)
E-cigarettes and Pipe/Water Pipes	25	3.7	0.01 (0.00–0.02)
E-cigarettes and Smokeless Tobacco Products	23	2.3	0.01 (0.00–0.01)
Cigars and Pipe/Water Pipes	24	8.0	0.02 (0.00–0.06)
Cigars and Smokeless Tobacco Products	20	0.3	0.001 (0.00–0.002)
Pipe/Water Pipes and Smokeless Tobacco Products	23	6.6	0.02 (0.00–0.05)

Specific tobacco product user group			

Group of smokers who use at least cigarettes	3,758	4,225.0	14.0 (13.4–14.6)
Cigarettes only	2,344	2,619.0	8.7 (8.1–9.3)
Multiple Products Use (Cigarettes and other tobacco product use)	1,414	1,606.0	5.3 (4.9–5.8)
Group of smokers who use at least HTPs	2,672	3,098.0	10.3 (9.7–10.8)
HTPs only	1,196	1,403.0	4.7 (4.3–5.1)
Multiple Products Use (HTPs and other tobacco product use)	1,476	1,695.0	5.6 (5.2–6.1)

E-cigarettes, electronic cigarettes; HTPs, heated tobacco products.

When estimates were 0 to the first decimal place, values are shown to the second decimal place.

^a^Use of tobacco products at baseline was defined as the use of tobacco products at least once in the past 30 days from the time of the baseline survey.

^b^Weight score (*N*, %) means estimated use of smoking products by smokers all over Japan using inverse probability-weighted scores calculated from a nationally representative cross-sectional survey. Note that the percentage of all categories combined does not equal 100%.

^c^Cigarettes include factory-made and roll-your-own cigarettes.

^d^HTPs include Ploom TECH, IQOS, and glo.

^e^E-cigarettes include e-cigarettes with/without nicotine, and e-cigarettes with unknown nicotine content.

^f^Smokeless tobacco products include chewing tobaccos and snus.

We show the results of the multivariate logistic regression, with non-smokers set as the baseline, in Table 3. The common factors between those positively associated with single-product users compared to non-smokers and those positively associated with MTP users compared to non-smokers included being male, having lower educational attainment, having severe psychological distress, having poor self-rated health, and alcohol use. However, when compared to non-smokers, age was a factor associated with both single-product users and MTP users, but in the opposite direction of relationship. For single-product users, compared to those aged 17–29 years, being older (30–59 years) was positively associated: 30–39 years (aOR 2.40; 95% CI, 1.92–2.98), 40–49 years (aOR 2.22; 95% CI, 1.82–2.71), and 50–59 years (aOR 1.93; 95% CI, 1.58–2.37). On the other hand, being 70–81 years old was negatively associated with single-product use (aOR 0.63; 95% CI, 0.48–0.82). For MTP users, compared to those aged 17–29 years, being 50 years or older was negatively associated: 50–59 years (aOR 0.65; 95% CI, 0.50–0.84), 60–69 years (aOR 0.44; 95% CI, 0.31–0.63), and 70–81 years (aOR 0.13; 95% CI, 0.09–0.19).

**Table 3.** Factors associated with MTP use and single-product use compared to non-smokers (*N* = 30,141)

		Single-product use	MTP use
		aOR (95% CI)^a^	aOR (95% CI)^a^
Age, years	17–29	REF	REF
	30–39	**2.40 (1.92–2.98)**	1.13 (0.87–1.48)
	40–49	**2.22 (1.82–2.71)**	0.94 (0.73–1.21)
	50–59	**1.93 (1.58–2.37)**	**0.65 (0.50–0.84)**
	60–69	1.07 (0.85–1.35)	**0.44 (0.31–0.63)**
	70–81	**0.63 (0.48–0.82)**	**0.13 (0.09–0.19)**
Sex	Female	REF	REF
	Male	**3.00 (2.65–3.38)**	**5.20 (4.19–6.44)**
Educational attainment	2 years of college or more	REF	REF
	High school or less	**1.40 (1.25–1.57)**	**1.53 (1.30–1.81)**
Psychological distress	None	REF	REF
	Moderate	0.96 (0.82–1.12)	1.05 (0.83–1.33)
	Severe	**1.03 (0.89–1.20)**	**2.01 (1.65–2.46)**
Self-rated health	Good	REF	REF
	Poor	**1.21 (1.02–1.44)**	**1.28 (1.04–1.57)**
Alcohol use	No/quit	REF	REF
	<2 times/week	**1.25 (1.07–1.46)**	**1.80 (1.38–2.36)**
	≥2 times/week	**1.74 (1.50–2.03)**	**3.19 (2.43–4.18)**

aOR, adjusted odds ratio; CI, confidence interval; MTP, multiple tobacco products; REF, reference.

Estimates were calculated using sampling weight from smoking products by smokers all over Japan.

A single-product user refers to a smoker who uses only one of the following tobacco products: cigarettes, heated tobacco products, e-cigarettes, cigars, pipe/water pipes, and smokeless tobacco products.

Boldface = statistically significant with *P* < 0.05.

^a^aOR was estimated by a multivariate logistic regression model adjusted with all variables simultaneously entered into the model.

We show the characteristics of all smokers who participated in the study in Table 4. Among smokers, 2,049 (32.8%) were MTP users. MTP users appeared to be associated with the following factors: younger age, male, severe psychological distress, heavy alcohol use, living with a smoker, exposure to tobacco company advertising in the last 6 months, current smoking rules in the workplace, and current smoking rules at home. The results of the Poisson regression analysis are also shown in Table 4. Among all smokers, the variables directly related to smoking that showed a positive association with MTP use were as follows. Living with a smoker was positively associated with MTP use (aPR 1.24; 95% CI, 1.09–1.42). Exposure to tobacco company advertising in the last 6 months was positively associated with MTP use (aPR 1.24; 95% CI, 1.10–1.39). Current smoking rules in the workplace were not associated with MTP use compared to having an indoor smoking ban in the workplace (partial smoking ban: aPR 0.92; 95% CI, 0.81–1.05 and no smoking ban: aPR 0.95; 95% CI, 0.70–1.29). Current loose smoking rules at home were positively associated with MTP use compared to those with a ban on both cigarettes and HTPs at home (both cigarettes and HTPs allowed at home: aPR 1.36; 95% CI, 1.15–1.61 and HTPs only allowed at home: aPR 1.73; 95% CI, 1.43–2.10).

**Table 4.** Factors associated with MTP use among smokers (*N* = 6,253)

		Single-product use (Total *N* = 4,204; 67.2%)	MTP use (Total *N* = 2,049; 32.8%)	*P* ^a^	MTP use
		Weighted *N* (%)	Weighted *N* (%)		aPR (95% CI)^b^
Age, years	17–29	575 (49.6)	584 (50.4)	**<0.001**	REF
	30–39	804 (66.8)	399 (33.2)		**0.70 (0.58–0.85)**
	40–49	1,039 (69.2)	463 (30.8)		**0.68 (0.58–0.81)**
	50–59	847 (74.3)	294 (25.8)		**0.59 (0.49–0.71)**
	60–69	572 (70.7)	237 (29.3)		**0.69 (0.54–0.89)**
	70–81	367 (83.4)	73 (16.6)		**0.43 (0.30–0.60)**
Sex	Female	1,219 (75.7)	392 (24.3)	**<0.001**	REF
	Male	2,986 (64.3)	1,657 (35.7)		**1.55 (1.33–1.82)**
Educational attainment	2 years of college or more	2,404 (67.1)	1,176 (32.9)	**<0.001**	REF
	High school or less	1,800 (67.3)	873 (32.7)		1.04 (0.92–1.16)
Psychological distress	None	2,324 (73.0)	857 (26.9)		REF
	Moderate	848 (71.0)	347 (29.1)	**<0.001**	1.05 (0.87–1.25)
	Severe	1,032 (55.0)	845 (45.0)		**1.38 (1.19–1.60)**
Self-rated health	Good	3,667 (67.6)	1,760 (32.4)		REF
	Poor	538 (65.1)	289 (35.0)	0.34	1.004 (0.86–1.17)
Alcohol use	No/quit	1,122 (74.1)	393 (25.9)		REF
	<2 times/week	1,261 (65.4)	665 (34.5)	**0.004**	1.11 (0.91–1.36)
	≥2 times/week	1,821 (64.8)	990 (35.2)		**1.30 (1.08–1.56)**
Living with a smoker	No	2,964 (70.3)	1,253 (29.7)	**<0.001**	REF
	Yes	1,240 (60.9)	796 (39.1)		**1.24 (1.09–1.42)**
Exposure to tobacco company advertising in the last 6 months	No	2,944 (71.0)	1,200 (29.0)	**<0.001**	REF
	Yes	1,261 (59.8)	849 (40.2)		**1.24 (1.10–1.39)**
Current smoking rules in the workplace	Indoor smoking ban	887 (61.1)	566 (39.0)	**<0.001**	REF
	Partial smoking ban (smoking room or smoking corner)	1,985 (67.1)	972 (32.9)		0.92 (0.81–1.05)
	No smoking ban	242 (66.9)	120 (33.1)		0.95 (0.70–1.29)
	Do not know	1,091 (73.6)	391 (26.4)		0.87 (0.71–1.07)
Current smoking rules at home	Both cigarettes and HTPs prohibited	1,205 (75.2)	397 (24.8)	**<0.001**	REF
	Both cigarettes and HTPs allowed	1,875 (66.5)	944 (33.5)		**1.36 (1.15–1.61)**
	HTPs only allowed	520 (53.3)	456 (46.7)		**1.73 (1.43–2.10)**
	Do not know	604 (70.6)	251 (29.4)		1.19 (0.91–1.55)

aPR, adjusted prevalence ratio; CI, confidence interval; MTP, multiple tobacco products; REF, reference.

All *N* and % were calculated using sampling weight from smoking products by smokers all over Japan.

A single-product user refers to a smoker who uses only one of the following tobacco products: cigarettes, heated tobacco products, e-cigarettes, cigars, pipe/water pipes, and smokeless tobacco products.

All weighted values were rounded.

Boldface = statistically significant with *P* < 0.05.

^a^χ2 test.

^b^aPR was estimated by a multivariate Poisson regression model adjusted with all variables simultaneously entered into the model.

## DISCUSSION

### The prevalence of MTP use in Japan in 2022

According to a JASTIS survey in 2017, 2 years after HTPs were introduced to the market in Japan, the prevalence of MTP use was 3.2%.^10^ From our findings, by 2022, this prevalence had risen to 6.8%, more than doubling within 5 years. Given the 2022 adult population in Japan (age 20 and over) was approximately 102 million,^19^ the number of MTP users is estimated to be roughly 6.9 million.

Our results showed that 32.8% of those using all types of tobacco products, 37.8% of those using at least cigarettes, and 54.3% of those using at least HTPs were MTP users in Japan (Table 2). The most common combination of dual use was cigarettes and HTPs, which accounted for 47.0% of MTP use. According to previous studies, HTPs use is a risk factor for MTP use,^20^^,^^21^ and younger age has been clarified as a risk factor for using HTPs.^15^ Thus, younger smokers are more likely to become MTP users than older smokers. Considering the evidence on the difficulty of quitting smoking for MTPs^22^ and HTPs^23^^,^^24^ users, young Japanese smokers may experience prolonged exposure to smoking-related health risks.

Our results also showed a 6.7% difference between the prevalence of cigarette smoking (14.0%) and the prevalence of using all types of tobacco products (20.7%), including cigarettes, HTPs, e-cigarettes, cigars, pipe/water pipes, and smokeless tobacco products (shown in Table 2). In considering future tobacco control measures, the government needs to continuously monitor not only the prevalence of cigarette smoking but also the prevalence of other tobacco products, including HTPs. HTPs were found to be the second most prevalent tobacco products (10.3%) in this study (shown in Table 2 and eTable 1).

According to recent CSLCPHW results, the smoking rate in Japan was 16.7% in 2019,^25^ which was relatively close to our estimate. This is thought to be because the set of covariates used to calculate inverse probability-weighted scores were adjusted for the responses of study participants, resulting in an estimate closer to that of the general population.^26^ Adjusting the obtained prevalence based on the weighted scores may still be effective for estimating the prevalence of use, including various tobacco products, because the use of HTPs and e-cigarettes is strongly associated.^27^^,^^28^

### Factors associated with single-product users compared to non-smokers

We clarified several factors associated with single-product users compared to non-smokers. Few studies focus on the associated factors of single-product users, but previous studies related to smoking are consistent with our findings. Smoking is associated with middle age,^29^ being male,^14^ low education,^30^ poor mental health,^14^^,^^31^ poor self-rated health,^32^ and alcohol use.^33^ As shown in Table 2, cigarettes are the most commonly used tobacco product among single-product users, followed by HTPs. However, as shown in eTable 1, HTPs constitute the largest proportion of single-product users among younger generations. One reason why HTPs are popular among the younger generation is the misconception that they are healthier than cigarettes.^34^ Single-product users may find it easier to quit smoking. However, a previous study has shown that HTP-only users have a risk of relapsing to cigarette use within 1 year.^35^ Therefore, it is important to provide smoking cessation support programs that focus on the use of both cigarettes and HTPs, especially for young single-product users.

### Factors associated with multiple tobacco product users compared to non-smokers

We clarified several factors associated with MTP users compared to non-smokers. Younger age,^8^ being male,^36^ and alcohol use^36^ are consistent with the results of previous studies focusing on MTP users. Low education and poor mental health were factors associated with MTP use. A review paper on MTP has pointed out a lack of studies clarifying the relationship between these factors and MTP use.^37^ Considering that MTP use leads to frequent smoking behavior, these associations can be explained by previous studies that have shown the correlation between low education,^30^ poor mental health,^31^ and smoking. The association between poor self-rated health and MTP use differed from the findings of a previous study.^10^ However, the results of our analysis can be explained by the following reasons: smokers generally have poor self-rated health^32^ and, in Japan, considering that many MTP users are also HTPs users, it is possible that in 2017, when the previous study^10^ was conducted, there had not yet been sufficient accumulation of exposure from MTP use. Therefore, as the history of MTP use lengthened, a deterioration in self-rated health may have been observed.

Our analysis found that, unlike single-product users, younger age was a an associated factor for MTP users compared to non-smokers. These results are supported by eTable 1, which shows that MTP use is more common among younger age groups. The previous study has shown that younger age is associated with the use of new tobacco products.^34^ The usage patterns of MTPs within the population change whenever a new tobacco product is released. Consequently, the health effects of MTP use might also change. Since not all MTP users use the same tobacco products, it is necessary to analyze their characteristics continuously using long-term follow-up data.

### Factors associated with multiple tobacco product users among smokers

We clarified several factors associated with a high prevalence of MTP use among smokers as follows: younger age, male, severe psychological distress, ≥2 times/week alcohol use, living with a smoker, exposure to tobacco company advertising in the last 6 months, and current smoking rules at home that allow the use of HTPs only and use of both cigarettes and HTPs.

While smoking rules at home were a associated factor of MTP use, smoking rules in the workplace were not. A previous study before the coronavirus disease 2019 pandemic reported that no workplace indoor smoking ban status was associated with a high prevalence of MTP use,^10^ which was inconsistent with the results of this study. However, a previous study conducted during the pandemic reported that workplace smoking rules were not associated with smoking cessation,^15^ which was consistent with the results of this study. The pandemic may have limited the effectiveness of workplace smoking rules on MTP due to increased opportunities to work from home.^38^

Current loose smoking rules at home, allowing the use of both cigarettes and HTPs, and allowing the use of HTPs only, were associated with a high prevalence of MTP use compared to a smoking ban on both cigarette and HTPs. Our findings were consistent with previous studies before the pandemic that found that a current smoking ban at home was negatively associated with cigarette^39^ and other tobacco product^40^^,^^41^ use. Implementing the current smoke-free rules at home might still effectively reduce MTP use.

### Strength and limitations

The strength of our study is that our survey data covers almost all smoking devices used in Japan. Conducting this survey on data from an ongoing internet survey allowed us to show the clear increase in MTP users between 2017 and 2022.

There are several limitations to this study. First, the data was collected through an internet survey, which may limit the generalizability of the results. However, the reported prevalence of smoking and the usage rates of various types of smoking products in Japan were calculated after adjustment for national representation using sampling weights. This allowed us to present one basic data set on the usage rates of various types of smoking products in Japan. Second, for non-popular products (eg, smokeless tobacco), the prevalence is very low, and the sample size was limited, so valid estimates might not be available.

### Conclusion

The study offers an understanding of the use of MTP in Japan and enables tracking of future patterns. In Japan, with an increase in HTP use, the number of MTP users has doubled in the past 5 years. This study contributes to the understanding of MTP use in Japan and enables tracking of future patterns. The most common combination of dual use was cigarettes and HTPs, accounting for 47.0% of MTP use. The current home smoking ban was found to help prevent MTP use, and this might be an effective countermeasure to reduce MTP use in the future.

## SUPPLEMENTARY MATERIAL

eTable 1. Prevalence of current use of various tobacco products among study participants (N = 5,519)
