# Supplementary material for: Estimating the Prevalence of and Clarifying Factors Associated With Multiple Tobacco Product Use in Japan: A Cross-sectional Study in 2022
Source: J Epidemiol. 2025 May 5;35(5):222–9. doi: 10.2188/jea.JE20240153 (PMC11979349; doi:10.2188/jea.JE20240153)
Supplement: Supplementary file 1 [file je-35-222-s001.pdf]

eTable 1. Prevalence of current use of various tobacco products among study participants (N=5,519)

Number of products used		Total tobacco user (N=5,519) N,% [a]	Age, years 17–29 N (%)	30–39 N (%)	40–49 N (%)	50–59 N (%)	60–69 N (%)	70–81 N (%)	Sex Male N (%)	Female N (%)
1	Cigarettes [b]	2,344 (42.5)	200 (8.5)	240 (10.2)	536 (22.9)	581 (24.8)	472 (20.1)	315 (13.4)	1,611 (68.7)	733 (31.3)
1	HTPs [c]	1,196 (21. 7)	176 (14.7)	227 (19.0)	338 (28.3)	245 (20.5)	163 (13.6)	47 (3.9)	841 (70.3)	355 (29.7)
1	E-cigarette [d]	87 (1.6)	22 (25.3)	12 (13.8)	19 (21.8)	20 (23.0)	9 (10.3)	5 (5.7)	63 (72.4)	24 (27.6)
1	Cigars	56 (1.01)	8 (14.3)	5 (8.9)	12 (21.4)	13 (23.2)	13 (23.2)	5 (8.9)	44 (78.6)	12 (21.4)
1	Pipe/Water Pipes	40 (0.7)	23 (57.5)	5 (12.5)	4 (10.0)	2 (5.0)	1 (2.5)	5 (12.5)	20 (50.0)	20 (50.0)
1	Smokeless Tobacco Products [e]	18 (0.3)	8 (44.4)	2 (11.1)	2 (11.1)	2 (11.1)	1 (5.6)	3 (16.7)	11 (61.1)	7 (38.9)
1	Total	3,741 (67. 8)	437 (11.7)	491 (13.1)	911 (24.4)	863 (23.1)	659 (17.6)	380 (10.2)	2,590 (69.2)	1,151 (30.8)
2	Cigarettes and HTPs	798 (14.5)	124 (15.5)	136 (17.0)	202 (25.3)	170 (21.3)	128 (16.0)	38 (4.8)	626 (78.4)	172 (21.6)
2	Cigarettes and E-cigarette	69 (1.3)	7 (10.1)	7 (10.1)	12 (17.4)	20 (29.0)	15 (21.7)	8 (11.6)	50 (72.5)	19 (27.5)
2	Cigarettes and Cigars	114 (2.1)	7 (6.1)	14 (12.3)	21 (18.4)	30 (26.3)	23 (20.2)	19 (16.7)	91 (79.8)	23 (20.2)
2	Cigarettes and Pipe/Water Pipes	18 (0.3)	7 (38.9)	1 (5.6)	2 (11.1)	3 (16.7)	2 (11.1)	3 (16.7)	13 (72.2)	5 (27.8)
2	Cigarettes and Smokeless Tobacco Products	6 (0.1)	0 (0)	0 (0)	1 (16.7)	3 (50.0)	0 (0)	2 (33.3)	5 (83.3)	1 (16.7)
2	HTPs and E-cigarette	107 (1.9)	19 (17.8)	13 (12.1)	31 (29.0)	18 (16.8)	18 (16.8)	8 (7.5)	76 (71.0)	31 (29.0)
2	HTPs and Cigars	25 (0.5)	5 (20.0)	3 (12.0)	6 (24.0)	3 (12.0)	6 (24.0)	2 (8.0)	17 (68.0)	8 (32.0)
2	HTPs and Pipe/Water Pipes	23 (0.4)	12 (52.2)	8 (34.8)	2 (8.7)	0 (0)	1 (4.3)	0 (0)	13 (56.5)	10 (43.5)
2	HTPs and Smokeless Tobacco Products	19 (0.3)	8 (42.1)	1 (5.3)	2 (10.5)	5 (26.3)	3 (15.8)	0 (0)	17 (89.5)	2 (10.5)
2	E-cigarette and Cigars	10 (0.2)	4 (40.0)	1 (10.0)	1 (10.0)	4 (40.0)	0 (0)	0 (0)	7 (70.0)	3 (30.0)
2	E-cigarette and Pipe/Water Pipes	7 (0.1)	7 (100)	0 (0)	0 (0)	0 (0)	0 (0)	0 (0)	4 (57.1)	3 (42.9)
2	E-cigarette and Smokeless Tobacco Products	4 (0.1)	4 (100)	0 (0)	0 (0)	0 (0)	0 (0)	0 (0)	2 (50.0)	2 (50.0)
2	Cigars and Pipe/Water Pipes	5 (0.1)	1 (20.0)	1 (20.0)	1 (20.0)	1 (20.0)	0 (0)	1 (20.0)	4 (80.0)	1 (20.0)
2	Cigars and Smokeless Tobacco Products	1 (0.02)	0 (0)	1 (100)	0 (0)	0 (0)	0 (0)	0 (0)	1 (100)	0 (0)
2	Pipe/Water Pipes and Smokeless Tobacco Products	4 (0.1)	3 (75.0)	1 (25.0)	0 (0)	0 (0)	0 (0)	0 (0)	3 (75.0)	1 (25.0)
2	Total	1,210 (21.9)	208 (17.2)	187 (15.5)	281 (23.2)	257 (21.2)	196 (16.2)	81 (6.7)	929 (76.8)	281 (23.2)
3	Cigarettes, HTPs, and E-cigarettes	93 (1.7)	27 (29.0)	17 (18.3)	19 (20.4)	15 (16.1)	13 (14.0)	2 (2.2)	73 (78.5)	20 (21.5)
3	Cigarettes, HTPs, and Cigars	67 (1.2)	11 (16.4)	6 (9.0)	17 (25.4)	17 (25.4)	15 (22.4)	1 (1.5)	52 (77.6)	15 (22.4)
3	Cigarettes, HTPs, and Pipe/Water Pipes	19 (0.3)	10 (52.6)	4 (21.1)	2 (10.5)	2 (10.5)	1 (5.3)	0 (0)	13 (68.4)	6 (31.6)
3	Cigarettes, HTPs, and Smokeless Tobacco Products	5 (0.1)	0 (0)	0 (0)	0 (0)	4 (80.0)	1 (20.0)	0 (0)	5 (100)	0 (0)
3	Cigarettes, E-cigarettes, and Cigars	6 (0.1)	0 (0)	2 (33.3)	1 (16.7)	2 (33.3)	1 (16.7)	0 (0)	6 (100)	0 (0)
3	Cigarettes, E-cigarettes, and Pipe/Water Pipes	2 (0.04)	1 (50.0)	1 (50.0)	0 (0)	0 (0)	0 (0)	0 (0)	1 (50.0)	1 (50.0)
3	Cigarettes, E-cigarettes, and Smokeless Tobacco Products	3 (0.1)	2 (66.7)	0 (0)	0 (0)	1 (33.3)	0 (0)	0 (0)	1 (33.3)	2 (66.7)
3	Cigarettes, Cigars, and Pipe/Water Pipes	17 (0.3)	0 (0)	1 (5.9)	3 (17.6)	4 (23.5)	4 (23.5)	5 (29.4)	16 (94.1)	1 (5.9)
3	Cigarettes, Cigars, and Smokeless Tobacco Products	4 (0.1)	2 (50.0)	1 (25.0)	1 (25.0)	0 (0)	0 (0)	0 (0)	2 (50.0)	2 (50.0)
3	Cigarettes, Pipe/Water Pipes, and Smokeless Tobacco Products	1 (0.02)	1 (100)	0 (0)	0 (0)	0 (0)	0 (0)	0 (0)	1 (100)	0 (0)
3	Cigars, Pipe/Water Pipes, and Smokeless Tobacco Products	1 (0.02)	0 (0)	0 (0)	1 (100)	0 (0)	0 (0)	0 (0)	1 (100)	0 (0)
3	E-cigarettes, Pipe/Water Pipes, and Smokeless Tobacco Products	6 (0.1)	6 (100)	0 (0)	0 (0)	0 (0)	0 (0)	0 (0)	1 (16.7)	5 (83.3)
3	E-cigarettes, Cigars, and Smokeless Tobacco Products	5 (0.1)	3 (60.0)	0 (0)	2 (40.0)	0 (0)	0 (0)	0 (0)	3 (60.0)	2 (40.0)
3	E-cigarettes, Cigars, and Pipe/Water Pipes	3 (0.1)	3 (100)	0 (0)	0 (0)	0 (0)	0 (0)	0 (0)	2 (66.7)	1 (33.3)
3	HTPs, Pipe/Water Pipes, and Smokeless Tobacco Products	13 (0.2)	9 (69.2)	2 (15.4)	1 (7.7)	1 (7.7)	0 (0)	0 (0)	9 (69.2)	4 (30.8)
3	HTPs, Cigars, and Smokeless Tobacco Products	12 (0.2)	7 (58.3)	2 (16.7)	1 (8.3)	0 (0)	1 (8.3)	1 (8.3)	11 (91.7)	1 (8.3)
3	HTPs, Cigars, and Pipe/Water Pipes	8 (0.1)	6 (75.0)	2 (25.0)	0 (0)	0 (0)	0 (0)	0 (0)	6 (75.0)	2 (25.0)
3	HTPs, E-cigarettes, and Smokeless Tobacco Products	6 (0.1)	5 (83.3)	0 (0)	0 (0)	1 (16.7)	0 (0)	0 (0)	6 (100)	0 (0)
3	HTPs, E-cigarettes, and Pipe/Water Pipes	15 (0.3)	11 (73.3)	1 (6.7)	2 (13.3)	1 (6.7)	0 (0)	0 (0)	10 (66.7)	5 (33.3)

3	HTPs, E-cigarettes, and Cigars	14 (0.3)	6 (42.9)	5 (35.7)	2 (14.3)	1 (7.1)	0 (0)	0 (0)	10 (71.4)	4 (28.6)
3	Total	300 (5.4)	110 (36.7)	44 (14.7)	52 (17.3)	49 (16.3)	36 (12.0)	9 (3.0)	229 (76.3)	71 (23.7)
4	Cigarettes, HTPs, E-cigarettes, and Cigars	21 (0.4)	10 (47.6)	4 (19.0)	3 (14.3)	2 (9.5)	2 (9.5)	0 (0)	16 (76.2)	5 (23.8)
4	Cigarettes, HTPs, E-cigarettes, and Pipe/Water Pipes	13 (0.2)	6 (46.2)	3 (23.1)	2 (15.4)	2 (15.4)	0 (0)	0 (0)	11 (84.6)	2 (15.4)
4	Cigarettes, HTPs, E-cigarettes, and Smokeless Tobacco Products	8 (0.1)	4 (50.0)	3 (37.5)	0 (0)	1 (12.5)	0 (0)	0 (0)	7 (87.5)	1 (12.5)
4	Cigarettes, HTPs, Cigars, and Pipe/Water Pipes	14 (0.3)	9 (64.3)	1 (7.1)	1 (7.1)	3 (21.4)	0 (0)	0 (0)	13 (92.9)	1 (7.1)
4	Cigarettes, HTPs, Cigars, and Smokeless Tobacco Products	6 (0.1)	1 (16.7)	4 (66.7)	1 (16.7)	0 (0)	0 (0)	0 (0)	6 (100)	0 (0)
4	Cigarettes, HTPs, Pipe/Water Pipes, and Smokeless Tobacco Products	9 (0.2)	4 (44.4)	1 (11.1)	2 (22.2)	2 (22.2)	0 (0)	0 (0)	7 (77.8)	2 (22.2)
4	Cigarettes, E-cigarettes, Cigars, and Pipe/Water Pipes	4 (0.1)	1 (25.0)	0 (0)	1 (25.0)	0 (0)	1 (25.0)	1 (25.0)	3 (75.0)	1 (25.0)
4	Cigarettes, E-cigarettes, Cigars, and Smokeless Tobacco Products	1 (0.02)	0 (0)	0 (0)	0 (0)	1 (100)	0 (0)	0 (0)	1 (100)	0 (0)
4	Cigarettes, E-cigarettes, Pipe/Water Pipes, and Smokeless Tobacco Products	0 (0)	0 (0)	0 (0)	0 (0)	0 (0)	0 (0)	0 (0)	0 (0)	0 (0)
4	Cigarettes, Cigars, Pipe/Water Pipes, and Smokeless Tobacco Products	4 (0.1)	1 (25.0)	2 (50.0)	0 (0)	1 (25.0)	0 (0)	0 (0)	4 (100)	0 (0)
4	E-cigarettes, Cigars, Pipe/Water Pipes, and Smokeless Tobacco Products	5 (0.1)	3 (60.0)	2 (40.0)	0 (0)	0 (0)	0 (0)	0 (0)	4 (80.0)	1 (20.0)
4	HTPs, Cigars, Pipe/Water Pipes, and Smokeless Tobacco Products	4 (0.1)	3 (75.0)	0 (0)	1 (25.0)	0 (0)	0 (0)	0 (0)	4 (100)	0 (0)
4	HTPs, E-cigarettes, Pipe/Water Pipes, and Smokeless Tobacco Products	16 (0.3)	13 (81.3)	2 (12.5)	1 (6.3)	0 (0)	0 (0)	0 (0)	15 (93.8)	1 (6.3)
4	HTPs, E-cigarettes, Cigars, and Smokeless Tobacco Products	8 (0.1)	4 (50.0)	3 (37.5)	1 (12.5)	0 (0)	0 (0)	0 (0)	5 (62.5)	3 (37.5)
4	HTPs, E-cigarettes, Cigars, and Pipe/Water Pipes	10 (0.2)	6 (60.0)	2 (20.0)	2 (20.0)	0 (0)	0 (0)	0 (0)	7 (70.0)	3 (30.0)
4	Total	123 (2.2)	65 (52.9)	27 (21.2)	15 (12.2)	12 (9.8)	3 (2.4)	1 (0.8)	103 (83.7)	20 (16.3)
5	Cigarettes, HTPs, E-cigarettes, Cigars, and Pipe/Water Pipes	18 (0.3)	6 (33.3)	4 (22.2)	5 (27.8)	2 (11.1)	1 (5.6)	0 (0)	17 (94.4)	1 (5.6)
5	Cigarettes, HTPs, E-cigarettes, Cigars, and Smokeless Tobacco Products	13 (0.2)	7 (53.8)	1 (7.7)	4 (30.8)	0 (0)	0 (0)	1 (7.7)	7 (53.8)	6 (46.2)
5	Cigarettes, HTPs, E-cigarettes, Pipe/Water Pipes, and Smokeless Tobacco Products	9 (0.2)	6 (66.7)	0 (0)	2 (22.2)	1 (11.1)	0 (0)	0 (0)	6 (66.7)	3 (33.3)
5	Cigarettes, HTPs, Cigars, Pipe/Water Pipes, and Smokeless Tobacco Products	11 (0.2)	6 (54.5)	2 (18.2)	1 (9.1)	1 (9.1)	1 (9.1)	0 (0)	8 (72.7)	3 (27.3)
5	Cigarettes, E-cigarettes, Cigars, Pipe/Water Pipes, and Smokeless Tobacco Products	2 (0.04)	1 (50.0)	0 (0)	0 (0)	1 (50.0)	0 (0)	0 (0)	2 (100)	0 (0)
5	HTPs, E-cigarettes, Cigars, Pipe/Water Pipes, and Smokeless Tobacco Products	33 (0.6)	17 (51.5)	4 (12.1)	8 (24.2)	3 (9.1)	1 (2.5)	0 (0)	25 (75.8)	8 (24.2)
5	Total	86 (1.6)	43 (50.0)	11 (12.8)	20 (23.3)	8 (9.3)	3 (3.5)	1 (1.2)	65 (75.6)	21 (24.4)
6	All	59 (1.1)	25 (42.4)	12 (20.3)	15 (25.4)	5 (8.5)	2 (3.4)	0 (0)	43 (72.9)	16 (27.1)

HTPs, heated tobacco products; e-cigarettes, electronic cigarettes.

[a] The percentage in total (n, %) indicates the column ratio.

[b] Cigarettes include factory-made and roll-your-own cigarettes.

[c] HTPs include Ploom TECH, IQOS, and glo.

[d] E-cigarettes include e-cigarettes with/without nicotine, and e-cigarettes with unknown nicotine.

[e] Smokeless tobacco products include chewing tobaccos and snus.
